# Factors Associated with Sexual Violence against Men Who Have Sex with Men and Transgendered Individuals in Karnataka, India

**DOI:** 10.1371/journal.pone.0031705

**Published:** 2012-03-20

**Authors:** Souradet Y. Shaw, Robert R. Lorway, Kathleen N. Deering, Lisa Avery, H. L. Mohan, Parinita Bhattacharjee, Sushena Reza-Paul, Shajy Isac, Banadakoppa M. Ramesh, Reynold Washington, Stephen Moses, James F. Blanchard

**Affiliations:** 1 Department of Community Health Sciences, Faculty of Medicine, Centre for Global Public Health, University of Manitoba, Winnipeg, Manitoba, Canada; 2 BC Centre for Excellence in HIV/AIDS, Vancouver, British Columbia, Canada; 3 School of Population and Public Health, University of British Columbia, Vancouver, British Columbia, Canada; 4 Karnatka Health Promotion Trust, Rajajinagar, Bangalore, India; Vanderbilt University, United States of America

## Abstract

**Objectives:**

There is a lack of information on sexual violence (SV) among men who have sex with men and transgendered individuals (MSM-T) in southern India. As SV has been associated with HIV vulnerability, this study examined health related behaviours and practices associated with SV among MSM-T.

**Design:**

Data were from cross-sectional surveys from four districts in Karnataka, India.

**Methods:**

Multivariable logistic regression models were constructed to examine factors related to SV. Multivariable negative binomial regression models examined the association between physician visits and SV.

**Results:**

A total of 543 MSM-T were included in the study. Prevalence of SV was 18% in the past year. HIV prevalence among those reporting SV was 20%, compared to 12% among those not reporting SV (p = .104). In multivariable models, and among sex workers, those reporting SV were more likely to report anal sex with 5+ casual sex partners in the past week (AOR: 4.1; 95%CI: 1.2–14.3, p = .029). Increased physician visits among those reporting SV was reported only for those involved in sex work (ARR: 1.7; 95%CI: 1.1–2.7, p = .012).

**Conclusions:**

These results demonstrate high levels of SV among MSM-T populations, highlighting the importance of integrating interventions to reduce violence as part of HIV prevention programs and health services.

## Introduction

Internationally, sexual violence has been recognized as a human rights priority, with significant implications for public health policy [1]. Sexual violence, according to the World Health Organization (WHO) is defined as “…any sexual act, attempt to obtain a sexual act, unwanted sexual comments or advances, or acts to traffic, or otherwise directed, against a person's sexuality using coercion, by any person regardless of their relationship to the victim, in any setting, including but not limited to home and work.”(p.149) [1] The potential negative health implications of experiencing sexual violence have been demonstrated; short- and long-term impacts of experiencing sexual violence include increased susceptibility to HIV and other sexually transmitted infections (STIs), physical and mental disability (e.g. depression and post-traumatic stress disorder), and mortality [2], [3], [4].

As most research has focused on women, sexual violence in other populations, such as men who have sex with men and transgendered individuals (MSM-T) has been largely overlooked, despite the demonstrated vulnerability of MSM-T to violence [1], [5]. MSM-T in developing countries, such as India, may be at heightened vulnerability, given strongly entrenched societal and structural sanctions against same-sex relationships [6], [7]. Correspondingly, a few studies have demonstrated that MSM-T in India have an elevated risk of experiencing violence [8], [9]. Prevalence of sexual violence, however, is likely to be under-reported, due to the stigma attached to reporting of sexual violence by men, as well as the marginalization of MSM-T communities in India [8]. Although MSM-T identity is highly nuanced and diverse in India [10], [11], [12], to varying degrees, stigma and discrimination have contributed to social and structural vulnerability [8]. For example, it was not until 2009 that ‘homosexual intercourse’ was decriminalised in a Delhi court [6], [13], overturning a discriminatory law that had been part of the Indian Penal Code since 1860 [6], [7]. Added to the ever-present stigma and discrimination against MSM-T, some MSM-T in India engage in commercial sex work [8], [10], raising the potential for these particular MSM-T to be at especially high risk, given the known vulnerabilities associated with sex work [14], [15], [16].

The social and structural vulnerability of MSM-T may be particularly problematic in the Indian context. Although the heterosexual spread of HIV, thought to be in large part through relationships between female sex workers (FSWs) and clients of FSWs, was an important factor in earlier phases of the HIV epidemic in southern India [17], [18], [19], [20], [21], heterogeneity in HIV transmission dynamics has increasingly been recognized [20], [22], [23], [24], [25], [26], [27], [28], [29], [30]. In particular, elevated rates of HIV among MSM-T have been reported in India [21], [31], [32], [33]. Despite this increasingly important role in HIV epidemiology, both in India and in other parts of South Asia [34], [35], [36], there has historically been a lack of information on MSM-T populations in India [8], [37].

Although recent studies have increased our understanding of MSM-T [26], [30], [38], [39], there is still little research examining sexual violence within MSM-T communities in South Asia. Using data from a cross-sectional study of four districts in Karnataka State, southern India, this study therefore sought to describe the prevalence of sexual violence among MSM-T, as well as examine factors related to experiencing violence. Given both the complexities related to violence against MSM-T in the Indian context, the demonstrated links between violence and HIV in other populations and the nuanced nature of MSM-T identities in India [40], the results can be used to inform future HIV prevention initiatives.

## Methods

### Study design and sampling

Institutional review boards at the University of Manitoba in Winnipeg, Canada and St. John's Medical College and Hospital in Bangalore, India approved the study and the verbal consent process. Interviews were conducted anonymously, with no names or personal identifiers recorded. Because of the high levels of marginalization and stigmatization of MSM-T populations in India, verbal consent was obtained for all respondents in lieu of written consent, as MSM-T are reluctant to sign their names to documents. An independent witness signed each consent form for the respondent, affirming that consent was correctly obtained. Data were from a cross-sectional behavioural and biological survey of MSM-T populations in four districts (sub-state administrative areas) in Karnataka State, southern India: Belgaum, Bellary, Mysore and Shimoga. Resources were only sufficient to conduct surveys in these four districts, plus an additional district, Bangalore Urban. Thus, the districts were chosen purposively, based on size of high risk populations and the socio-cultural regions of Karnataka [41]. MSM-T in Belgaum (n = 250), Bellary (n = 101), Mysore (n = 100) and Shimoga (n = 92) were recruited in 2008 through a multistage cluster sampling technique, as described in some depth by Saidel et al. [42], and in other previous published studies [38], [40]. Briefly, as per the IBBA protocol, a sample size of 400 at the district level was thought adequate to detect an absolute difference of 15% or more from an assumed value of 50% in key behavioural characteristics between survey administrations, with 95% confidence and 90% power [42]. The four districts in this analysis were treated as a single study, and the target sample size was inflated to 550 in order to ensure adequate representation. Selection of solicitation sites occurred in the first stage of sampling, and selection of MSM-T in the second. Time-location cluster sampling was used to select specific clusters. Informed by previous mapping exercises, a total of 110 clusters with a minimum 5 interviews in each cluster were targeted across the four districts. Within each cluster, MSM-T were randomly approached by field staff and asked to participate. Free transportation was then arranged to a private venue for those MSM-T agreeing to participate. At the venue, MSM-T were explained IBBA procedures in detail, and given the opportunity to ask questions; the voluntary nature of the survey was stressed, especially the ability of the participant to withdraw participation at any point in the survey. Prior to field work and recruitment, a specific effort was made to inform community members of the scope, purpose and the risks and benefits of the IBBA by field workers through community-based organizations.

Surveys were conducted as part of a baseline assessment of HIV prevention programs located in these districts. Program and mapping activities were directed at the most at- risk MSM-T [38], [40], and broadly focused on those frequenting cruising sites, and practicing receptive anal intercourse. The larger sample size of Belgaum reflects the larger MSM-T population in that district. Bangalore was omitted from the present analysis as MSM-T in Bangalore were surveyed over a different time frame than the other four districts. Participants were included in the study if they were 18 years or older, and reported having had sex with a man at least once in their lifetime.

### Survey organization and methods

MSM-T were interviewed individually through a structured questionnaire administered face-to-face by trained peer workers in the local language (e.g. individuals from within the MSM-T community). As in previous studies [41], biological data were gathered using blood and urine samples. HIV serological testing was conducted using Microelisa (J. Mitra and Company, India), and positive tests were confirmed using Genedia HIV 1/2 ELISA 3.0 (Green Cross Life Science Corporation, South Korea). Where serum samples were not provided, a dried blood spot was performed on finger prick blood using the same serological tests. When neither serum nor finger prick samples were provided, urine samples were tested for HIV by Calypte (Biomedical Corporation, Berkeley, California, USA), and positives were confirmed by Western blot. When confirmed by Western blot, urine samples have been shown to have 99% sensitivity [43], [44] and 100% specificity [43]. Chlamydia and gonorrhoea testing was done using the Gen-Probe Aptima assay (Gen-Probe Incorporated, San Diego, USA). Respondents could choose to answer the behavioural portion of the IBBA if they chose not to provide samples.

### Measures

Respondents were asked if they had been physically forced to have sexual intercourse with someone in the past year; those answering ‘yes’ were classified as having experienced sexual violence. Two measures of physician utilization were used as outcome variables: one measuring the number of non-specific physician visits, while the other asked about STI-related visits. Similar to the sexual violence question, the timeframe for both physician utilization questions was over the past year. Respondents were compared on socio-demographic, sexual behaviour, health care access and biological variables. Age, literacy levels, marital status, district of residence, and sexual identity were included as socio-demographic variables.

In India, sexual identity among MSM-T is highly diverse and nuanced, thus it should be noted that any categorization of MSM-T may not necessarily capture this complexity [12], [40]. Similar to other studies [38], MSM-T could self-identify as *Kothis* (those who primarily practice receptive anal sex); *Hijras* (transgenders who often self-identify as female); *Panthis* (those who primarily practice insertive anal sex and are often clients of Kothis or Hijras); *Double-deckers* (those who practice both insertive/receptive anal sex), and *others* [40]. For the purposes of this study, and balancing the need to create meaningful categories, we grouped MSM-T into the following identities: *Kothi/Hijras* (due to the heightened vulnerability of “feminine” MSM-T [8], [11], [26]); *Double-deckers* (as over 50% of the sample was comprised of this identity); and all others. As indicated above, there were relatively few *Panthis* in the sample, as they are not a focus of programmatic efforts. Age at first vaginal and anal intercourse, use of condoms generally and at last anal intercourse, reasons for not using condoms, usual place of solicitation of other MSM-T, usual place for anal sex, frequency of anal sex in the last week, and condom use with both regular and non-regular male sex partners, were used to characterize sexual behaviour. In addition to general sexual behaviour questions, respondents were asked whether or not they had a “primary” male sex partner. In terms of sex work–related questions, a positive answer to the question “Have you ever received cash or a gift in return for anal sex” was interpreted as having been involved in sex work; those answering positively to this question were asked at what age their first paid sex occurred.

### Statistical Analysis

For analyses examining the factors related to sexual violence, bivariate logistic regression models were used to test for significant associations between experiencing sexual violence and all variables of interest. Factors that were significantly associated with experiencing violence at the p<.10 level, using the adjusted Wald test, and that did not have more than 1% missing data, were included for multivariable analyses. For multivariable analyses, multiple logistic regression models were used to assess the impact of included variables on the odds of experiencing sexual violence. *A priori*, HIV status, district of residence and identity were thought to be important variables to adjust for, and thus these variables were forced into models. In addition to models using the entire sample, and because of the high levels of association between sex work, *kothi*-identity, and number of male sexual partners [8], multivariable models stratified by sex work were also fitted to the data.

As physician utilization was a count variable (i.e., the number of discrete physician visits), bivariate and multivariable negative binomial regression models were used for analyses examining the association between sexual violence and physician utilization [45]. Crude and adjusted relative risks (RR and ARR respectively) are reported for these analyses. For comparability purposes, the same set of variables used in examining the factors of sexual violence were adjusted for in the multivariable negative binomial models. Finally, models were stratified by sex work status. For all analyses, sampling weights were utilized in multiple regression models to account for the complex sampling design, using survey methods in Stata 11 [46]. Multicollinearity in multivariable models was assessed using the variance inflation factor (VIF) and tolerance statistics, corrected for the survey methods employed [47].

## Results

A total of 543 MSM-T were included in the study. All percentages, means and p-values reported in tables are based on the results from weighted analyses, and sample sizes reported are unweighted. The mean age of respondents was 31.3 years, with slightly less than half of the sample reporting being currently married (Table S1). At 0.7% and 0.4%, respectively, chlamydial and gonorrheal infections were rare, while 13.5% of the sample tested positive for HIV. In total, 17.5% of the sample reported experiencing sexual violence in the last year. The most common sources of violence were clients (61%), police (18%), a regular partner (13%), a pimp (10%) and ‘other’ (21%). The mean age of respondents for the entire sample was 30.9 years (SD: 9.5), with an inter-quartile range of 23–37, with slightly less than half of the sample reporting being currently married. MSM-T reporting violence tended to be younger, with the mean age of those reporting violence 27.7 years, compared to 31.6 for those not reporting violence; similarly, MSM-T reporting violence were less likely to be married (30.5% vs. 56.2%), and to self-identify as Kothi/*Hijra* (45.8% vs. 22.6%). MSM-T from Belgaum represented the largest majority of respondents, at 46% (n = 250).

### Bivariate Analyses

#### Prevalence of Sexual Violence by Socio-Demographic Characteristics

Table S2 examines the prevalence of violence by variables of interest. MSM-T who were currently married were less likely to have reported sexual violence (8.6% vs. 21.6%, p<.001), and those self-identifying as *Kothi/Hijra* reported the highest levels of sexual violence (25.9%, p<.001). The prevalence of sexual violence varied significantly by district (p = .036), with MSM-T from Mysore reporting the highest prevalence of sexual violence, at 31% compared to 12%, 13% and 22% in Shimoga, Belgaum, and Bellary, respectively. HIV prevalence was higher among MSM-T experiencing sexual violence (20.3% vs. 12.4%).

#### Prevalence of Sexual Violence by General Sexual Behaviour Characteristics

MSM-T who reported never having vaginal intercourse were more likely to report experiencing sexual violence (22.3% vs. 10.6%, p = .002); age at first vaginal intercourse was not significantly different among those reporting vaginal intercourse (p = .290). MSM-T experiencing sexual violence were younger at first anal intercourse (17.0 vs. 18.8 years, p = .029), and those reporting condom use in last anal intercourse were more likely to have experienced sexual violence (16.3% vs. 2.2%, p<.001). MSM-T experiencing violence reported having anal sex with a higher number of regular male sex partners (p = .088) and non-regular male sex partners (p = .043) in the past week.

#### Prevalence of Sexual Violence by Sex Work Characteristics & Health-Care Access Behaviours

Sexual violence prevalence was 27.2% for those reporting sex work, compared to 10.7% for those who did not (p<.001). On average, MSM-T experiencing sexual violence reported more general doctor visits in the past year (12.5 vs. 10.3 visits, p = .017). Although there were no statistically significant differences in ever having taken an HIV test (p = .690),

### Multivariable Analyses

Table S3 shows the results from multivariable analyses of variables for the total sample that were significant at the p<.10 level in bivariate analyses. The variables measuring number of regular/non-regular partners were highly correlated (r>.90); thus, given the more substantive interest in non-regular partners, only the variable measuring number of non-regular partners was included in multivariable models. Sexual violence was associated with younger age (AOR: 0.9, 95%CI: 0.9,1.0; p = .001); and *Kothi/Hijra* identity (AOR: 3.5, 95%CI: 1.2,10.6; p = .027). Having anal sex with 5+ non-regular male sex partners was marginally non-significant at the p<.05 level (AOR: 1.8, 95%CI: 1.0,3.5; p = .067). Upon stratification by sex work (Table 1), and for MSM-T reporting sex work, only having 5+ non-regular male partners in the last week was associated with sexual violence, adjusting for all other variables in the model (AOR: 4.1; 95%CI: 1.2,14.3; p = .029). For those not reporting sex work, *Kothi/Hijra* identity (AOR: 6.0; 95%CI: 1.3,28.8; p = .026) and age (AOR: 0.9; 95%CI: 0.9,1.0; p = .002) remained significantly associated with sexual violence (Table 1).

**Table 1:** Crude (OR) and Adjusted odds ratios (AOR) and 95% confidence intervals (95%CI) from weighted logistic regression models examining correlates of sexual violence among men who have sex with men and transgenders (MSM-T), stratified by sex work status, Karnataka, South India (2008)*.

		Violence Prevalence (%)	OR	95% CI	AOR	95% CI	P**
**Sex Work (N = 175)**							
Age (mean)		26.9	0.99	(0.94,1.03)	0.97	(0.95,1.06)	.276
Identity***	Bisexual/Panthi/Other	39.0	*Ref*	*–*	*Ref*	*–*	*–*
	Double decker	19.9	0.39	(0.05,3.28)	1.15	(0.17,7.79)	.884
	Kothi/Hijra	31.7	0.72	(0.10,5.48)	1.23	(0.17,8.76)	.834
Currently Married	No	29.7	*Ref*	*–*	*Ref*	*–*	*–*
	Yes	20.4	0.61	(0.25,1.49)	0.92	(0.32,2.67)	.881
Has main male sex partner	No	17.5	*Ref*	*–*	*Ref*	*–*	*–*
	Yes	35.2	2.56	(1.12,5.89)	1.82	(0.77,4.32)	.166
District	Belgaum	22.9	*Ref*	*–*	*Ref*	*–*	*–*
	Bellary	36.4	1.92	(0.71,5.20)	1.50	(0.48,4.75)	.477
	Shimoga	25.0	1.12	(0.28,4.56)	1.52	(0.36,6.33)	.558
	Mysore	30.1	1.45	(0.58,3.63)	1.61	(0.47,5.45)	.434
Non-regular male sex partners, past week	0	18.3	*Ref*	*–*	*Ref*	*–*	*–*
	1–4	26.9	1.64	(0.57,4.74)	1.57	(0.47,5.27)	.452
	5+	52.4	4.90	(1.58,15.17)	4.08	(1.17,14.26)	.029
HIV status	Negative	24.0	*Ref*	*–*	*Ref*	*–*	*–*
	Positive	40.8	2.19	(0.75,6.34)	2.44	(0.63,9.48)	.192
**No Sex Work (N = 368)**							
Age (mean)		27.4	0.93	(0.88,0.97)	0.92	(0.87,0.97)	.002
Identity***	Bisexual/Panthi/Other	4.3	*Ref*	*–*	*Ref*	*–*	*–*
	Double decker	10.3	2.58	(0.81,8.22)	2.73	(0.76,9.85)	.121
	Kothi/Hijra	19.0	5.25	(1.43,19.23)	6.00	(1.25,28.81)	.026
Currently Married	No	16.6	*Ref*	*–*	*Ref*	*–*	*–*
	Yes	7.0	0.38	(0.18,0.79)	1.02	(0.42,2.50)	.958
Has main male sex partner	No	9.0	*Ref*	*–*	*Ref*	*–*	*–*
	Yes	13.9	1.63	(0.75,3.55)	1.17	(0.49,2.80)	.715
District	Belgaum	10.3	*Ref*	*–*	*Ref*	*–*	*–*
	Bellary	13.5	1.37	(0.45,4.15)	1.01	(0.30,3.46)	.986
	Shimoga	8.9	0.86	(0.27,2.74)	0.66	(0.18,2.43)	.525
	Mysore	33.3	4.38	(1.84,10.45)	2.00	(0.71,5.69)	.187
Non-regular male sex partners, past week	0	10.2	*Ref*	*–*	*Ref*	*–*	*–*
	1–4	11.9	1.18	(0.54,2.57)	1.36	(0.60,3.08)	.455
	5+	4.0	0.37	(0.07,2.08)	0.29	(0.04,1.79)	.179
HIV positive status	Negative	10.5	*Ref*	*–*	*Ref*	*–*	*–*
	Positive	12.7	1.24	(0.28,5.61)	1.49	(0.31,7.24)	.614

* All percentages are weighted percentages;

** P-value reported for adjusted analyses only.

*** Kothis: those who primarily practice receptive anal sex; Hijras: transgenders who often self-identify as female; Panthis: those who primarily practice insertive anal sex; Double-deckers: those who practice both insertive/receptive anal sex.

Table 2 shows the results from analyses examining physician utilization, including only those individuals who provided complete answers to the utilization questions. On average, MSM experiencing sexual violence reported more physician visits, both for general (12.5 vs. 10.3) and for STI-related (1.7 vs. 1.5) reasons. In adjusted analyses, experiencing sexual violence was significantly associated with a 40% increase in general physician visits when the entire sample was examined (ARR: 1.4; 95%CI: 1.1,1.7; p = .012) and 70% in those reporting sex work (ARR: 1.7; 95%CI: 1.1,2.7 p = .012).

**Table 2:** Crude (RR) and adjusted relative risk ratios (ARR) and 95% confidence intervals (95%CI) from weighted negative binomial regression models examining association between general and sexually transmitted infections-related physician utilizations (past 1 year) and sexual violence among men who have sex with men and transgenders (MSM-T), by total sample and stratified by sex work status, Karnataka, South India (2008).

		Mean Utilization	RR	95% CI	ARR	95% CI	P value*
**General Physician Visits**	**Total Sample** **(N = 378)**						
	Did not experience violence	10.3	*Ref*	*–*	*Ref*	*–*	.012
	Experienced violence	12.5	1.30	(1.01,1.67)	1.35	(1.07,1.71)	
	**Reported Sex Work** **(N = 140)**						
	Did not experience violence	12.0	*Ref*	*–*	*Ref*	*–*	.012
	Experienced violence	16.6	1.39	(0.87,2.21)	1.74	(1.14,2.66)	
	**Did Not Report Sex Work (N = 238)**						
	Did not experience violence	9.8	*Ref*	*–*	*Ref*	*–*	.284
	Experienced violence	10.3	1.04	(0.74,1.49)	1.21	(0.85,1.73)	
**STI-Related Physician Visits**	**Total Sample** **(N = 376)**						
	Did not experience violence	1.5	*Ref*	*–*	*Ref*	*–*	.835
	Experienced violence	1.7	1.13	(0.63,2.03)	1.07	(0.55,2.09)	
	**Reported Sex Work** **(N = 140)**						
	Did not experience violence	2.5	*Ref*	*–*	*Ref*	*–*	.302
	Experienced violence	2.1	0.82	(0.33,2.02)	1.60	(0.64,3.99)	
	**Did Not Report Sex Work (N = 236)**						
	Did not experience violence	1.2	*Ref*	–	*Ref*	–	.160
	Experienced violence	1.4	1.14	(0.52,2.53)	0.58	(0.27,1.25)	

* P-value reported for adjusted analyses only; models adjusted for age, sexual identity, marital status, district, number of unknown partners, presence of a main sexual partner and HIV status.

## Discussion

This study demonstrated a high level of sexual violence among MSM-T, with nearly one out of every five MSM-T experiencing sexual violence in the year prior to their interview. This prevalence is less than reported by Newman et al., who found an overall prevalence of past year sexual violence of 41% in a sample of MSM-T from Chennai [5]. However, similar to our results, paid sex work was associated with higher rates of sexual violence, highlighting the increased risk of sexual violence to MSM-T who engage in sex work, over and above the already elevated rates of violence and harassment experienced by Indian MSM-T in general [8], [9]. Notably, the prevalence of sexual violence reported in our study is higher than the reported prevalence among FSWs in southern India, which has been estimated at 10–15% [48]. Our results suggest that an increased likelihood of sexual violence was associated with greater numbers of non-regular male sex partners, but only among those who reported an involvement in sex work. Among MSM-T who reported no involvement in sex work, older age was associated with decreasing risk, while the odds of having experienced sexual violence was over six-fold for those respondents identifying as *Kothis* or *Hijras*. Although in univariate analysis an association was found between condom use at last anal intercourse and increased sexual violence, it should be noted that this variable had 10% missing data; thus caution is warranted in interpretation of this result. As well, this association may be confounded by MSM-T who practice sex work generally having higher rates of condom use at last intercourse (96% vs. 90%, data not shown).

### Feminine identities and the risk of sexual violence

The observation of heightened risk of sexual violence among *Kothis* and *Hijras* aligns with previously published work [8]. In the Indian context, Chakrapani et al. have noted that the complex interplay between macro-level environments interact to create “…interlocking subsystems supporting direct and indirect victimization…”(p.358) [8] of MSM-T, and in particular, those MSM-T that self-identified as *Kothis* [8]. At the community level, *Kothis* are often intimidated for sex and money, while acts of harassment, extortion, and physical and sexual assault perpetrated by police have been reported. Given systematic discrimination at the health care level, *Kothi*-identified MSM-T are left little recourse for protection [8]. Ultimately, and with respect to HIV infection, this complex dynamic results in increased risk for MSM-T from multiple fronts: protective measures, such as condom use are difficult to negotiate in the context of sexual violence, while the medical system offers minimal support for those experiencing violence.

Like *Kothis*, *Hijras* (i.e., transgendered) have few available economic choices [11], [49]; however, as Reddy suggests, *Hijras*' highly visible nature, in combination with their particular lack of societal power, may lead to further stigma, marginalization and vulnerability [49]. HIV prevalence of nearly 50% among *Hijras* has been noted by other published studies [50]. Indeed, in our sample, *Hijras*, at 26%, had the highest HIV prevalence, followed by *Kothis*, *Panthis/Bisexuals/Others*, and *Double-deckers* (11%). Combined, HIV prevalence among *Kothis/Hijras* was 20%.

Given the complex and multi-level factors responsible for the continued discrimination of MSM-T, our findings suggest the importance of structural and societal changes to help alleviate the vulnerabilities associated with MSM-T identity in India. Decriminalization of ‘homosexual’ activities is one important step towards reducing discrimination against MSM-T. However, without concurrent interventions aimed at other societal institutions, such as the policing and medical subsystems, the impact of state-level decriminalization on the safety and well-being of MSM-T may be lessened. Finally, and recognizing, as Boyce does, that ascribing the interpretation of *Kothi* identities with “self-evident meanings and behavioural patterns” (p.178) [12] is itself limiting for the purposes of understanding MSM-T in South Asia, it is imperative to explore the reasons why risk for sexual violence is seemingly elevated for those who take on this identity. Given the discordance in social meanings around *Kothi* identities, occurring between how the term is used in practice and how *Kothi*-identifying men perceive themselves [12], [49], thoughtful examinations of whether the increased risk of sexual violence is due to how perpetrators perceive *Kothi*s (e.g. as being easier targets), how *Kothis* view themselves (e.g., powerless), or some combination of both, are much needed. Disentangling this fundamental association will be important for informing effective sexual violence prevention initiatives.

### Sex work and the risk of sexual violence

Although evidence is limited in the Indian MSM-T context, engaging in sex work has been associated with vulnerability to violence [5], [8]. As reported by Newman et al. in their study of MSM-T in Chennai, the proportion of MSM-T engaging in sex work was 68% among those experiencing sexual violence in the past year; in comparison, among those not experiencing sexual violence, this proportion was 54% [5]. In this same study, the authors found that MSM-T engaging in sex work were two-fold more likely to have experienced physical and verbal harassment in the past year. This aligns with our finding of a higher prevalence of sexual violence among MSM-T reporting sex work (28% vs. 10%). The situations contributing towards involvement of MSM-T in sex work, including environments of desperation and survival, can help to explain the elevated risk among those involved in sex work [51]. As Chakrapani et al. write, cruising in public sex environments for potential clients place MSM-T at higher risk of direct forms of violence from members of society (e.g., rowdies) and from those in authority (e.g., police). Moreover, the dual marginalization of MSM-T and sex work contribute to indirect forms of violence, as MSM-T are likely to be exploited because they cannot seek recourse against those who perpetrate direct forms of violence against them [8]. Although MSM-T often turn to sex work for survival reasons, heterogeneity in reasons behind becoming a sex worker, including those of desire and sexual expression should be recognized [10].

Irrespective of trajectories and reasons involved, it is clear that sex work places MSM-T at higher risk of sexual violence. An increased exposure to clients may be one reason why higher numbers of non-regular partners was associated with a greater risk for sexual violence in our study only among sex workers. Although representing a different marginalized population (and recognizing important limitations in their comparison), the experience of FSWs in India may be informative [52], [53], [54]. Because female sex work is quasi-criminalized, policing and enforcement policies can operate to increase risk for experiencing sexual or physical violence, particularly for sex workers in street-based or public settings. To avoid being arrested and/or having their clients arrested, research indicates that women in sex work in other settings are forced to work in more isolated settings where they are at higher risk for client violence and further away from health services [2], [54], [55], [56]. Similarly, the dual social marginalization experienced by MSM-T who do sex work in southern India may exacerbate the risk for different types of violence.

Our results also highlight the importance of including anti-violence components in HIV prevention programs for highly vulnerable MSM-T, as has been undertaken in southern India recently for FSWs [54]. Indeed, the longer history of MSM programming in Mysore may have impacted both the recognition, and reporting of sexual violence, thus contributing to the higher prevalence observed there [57]. It should be noted that given MSM-T's still-precarious position within Indian society [6], [13], and ours and others' demonstration of high levels of sexual violence, the urgency to develop and implement initiatives aimed at eliminating sexual violence cannot be overemphasized. At the same time, research is needed to better understand how best to address violence against MSM-T, particularly for MSM-T who do sex work, in prevention programs. Prospective studies examining HIV risk over time may help to inform the timing of prevention/intervention studies, and qualitative studies that delineate the specific intersecting vulnerabilities faced by MSM-T according to sexual identity and involvement in sex work are much needed.

Importantly, our observation of higher rates of general physician utilization among those experiencing sexual violence suggests those who experience sexual violence have a greater demand for health services. Therefore, developing clinical services integrated with sexual violence services could be stressed where sexual violence is common [58]. That interventions have been implemented in the four districts with a specific focus on health care access among high-risk MSM-T groups, such as *Kothis/Hijras* with high numbers of sex partners [59], suggests the importance of future studies evaluating the extent to which interventions have had an impact on utilization of health care. As the HIV epidemic across South Asia becomes further entrenched in MSM-T populations [21], [31], [32], [33], [60], the establishment of an effective interface between health care systems, and MSM-T and their subpopulations may impact the trajectory of the epidemic in this region. Studies characterising the unique risks and environments of MSM-T may be used to educate and inform practitioners and policy-makers, thus having wide-ranging implications in the policy response to HIV in this vulnerable population.

There were a number of limitations to the study; first and foremost, the study sample was relatively small, thus it was not possible to conduct district-specific analysis. The study may have been insufficiently powered to detect differences in HIV prevalence by sexual violence status. However, having data collected from four districts using a well-established sampling methodology ensured a wider spectrum of responses, with an approximation of representation of the wider population of MSM-T. At the same time, the substantial differences in socio-cultural and political environments across different states in India should not be underestimated; thus, inferences from our sample to other states/populations should be undertaken with caution. Second, IBBA sampling methodology primarily relied on sampling from cruising sites, therefore our findings may not be generalisable to those MSM-T who do not frequent cruising sites. This sampling strategy was employed however, on the rationale that MSM-T who frequented cruising sites were at the highest HIV risk. Third, sexual identity of MSM-T was necessarily simplified and broad; future studies with a larger sample size may be able to better distinguish among sexual identities. Fourth, sexual violence was also measured fairly broadly; at the minimum, future studies should include more specific questions on violence by partner type. Fifth, the possibility of recall bias exists, as all behavioural questions were self-reported; this may be especially important considering some of the time frames involved in the behavioural questions (e.g., age at first sexual intercourse, ever use of condoms with partners, etc.). As well, the accuracy of one-year recall of physician visits may be called into question; having a six-month assessment of physician visits may help bolster the findings from the present study. However, there is no reason to suspect a systematic bias for providing incorrect answers, based on socio-demographic characteristics, and thus, the bias would tend to render associations towards the null. Finally, data were of a cross-sectional nature, and thus causality could not be inferred from our study.

In conclusion, the high rate of sexual violence in this sample of MSM-T further illustrates the importance of understanding vulnerabilities associated with MSM-T identity in India. As protection of marginalized populations from sexual violence is considered a fundamental human rights priority, these results may be used to help guide intervention activities among MSM-T, particularly in subpopulations shown to be at especially high risk.

### ETHICS APPROVAL

Institutional review boards at the University of Manitoba in Winnipeg, Canada and St. John's Medical College and Hospital in Bangalore, India approved the study and the verbal consent process. Interviews were conducted anonymously, with no names or personal identifiers recorded. Because of the high levels of marginalization and stigmatization of MSM-T populations in India, verbal consent was obtained for all respondents in lieu of written consent, as MSM-T are reluctant to sign their names to documents. An independent witness signed each consent form for the respondent, affirming that consent was correctly obtained.

## Supporting Information

Table S1 **Comparison of socio-demographic, sexual behaviour and health care access characteristics among men who have sex with men and transgenders (MSM-T) experiencing sexual violence in the last year, Karnataka, South India (2008).**
(DOC)

Table S2 **Prevalence of violence by socio-demographic, sexual behaviour and health care access characteristics among men who have sex with men and transgenders (MSM-T) experiencing sexual violence in the last year, Karnataka, South India (2008).**
(DOC)

Table S3 **Crude (OR) and Adjusted odds ratios (AOR) and 95% confidence intervals (95%CI) from weighted logistic regression models examining correlates of sexual violence among men who have sex with men and transgenders (MSM-T), by total sample and stratified by sex work status, Karnataka, South India (2008).**
(DOC)

## References

[1] Krug EG, Dahlberg LL, Mercy JA, Zwi AB, Lozano R (2002). (2002) World Report on Violence and Health.

[2] Shannon K, Kerr T, Strathdee SA, Shoveller J, Montaner JS (2009). Prevalence and structural correlates of gender based violence among a prospective cohort of female sex workers.. BMJ.

[3] Dunkle KL, Jewkes RK, Brown HC, Gray GE, McIntryre JA (2004). Gender-based violence, relationship power, and risk of HIV infection in women attending antenatal clinics in South Africa.. Lancet.

[4] El-Bassel N, Gilbert L, Wu E, Go H, Hill J (2005). HIV and intimate partner violence among methadone-maintained women in New York City.. Soc Sci Med.

[5] Newman PA, Chakrapani V, Cook C, Shunmugam M, Kakinami L (2008). Correlates of paid sex among men who have sex with men in Chennai, India.. Sex Transm Infect.

[6] Skanland CA (2009). India: Delhi high court annuls law criminalizing adult homosexual relations.. HIV AIDS Policy Law Rev.

[7] Misra G (2009). Decriminalising homosexuality in India.. Reprod Health Matters.

[8] Chakrapani V, Newman PA, Shunmugam M, McLuckie A, Melwin F (2007). Structural violence against Kothi-identified men who have sex with men in Chennai, India: a qualitative investigation.. AIDS Educ Prev.

[9] Safren SA, Martin C, Menon S, Greer J, Solomon S (2006). A survey of MSM HIV prevention outreach workers in Chennai, India.. AIDS Educ Prev.

[10] Lorway R, Reza-Paul S, Pasha A (2009). On becoming a male sex worker in Mysore: sexual subjectivity, “empowerment,” and community-based HIV prevention research.. Med Anthropol Q.

[11] Nanda S (1999). Neither Man nor Woman.

[12] Boyce P (2007). ‘Conceiving kothis’: men who have sex with men in India and the cultural subject of HIV prevention.. Med Anthropol.

[13] Baijal P, Kort R (2009). XVII International AIDS Conference: From Evidence to Action - Regional focus.. J Int AIDS Soc.

[14] Ward H, Aral SO (2006). Globalisation, the sex industry, and health.. Sex Transm Infect.

[15] Ward H, Day S, Mezzone J, Dunlop L, Donegan C (1993). Prostitution and risk of HIV: female prostitutes in London.. BMJ.

[16] Rekart ML (2005). Sex work harm reduction.. Lancet.

[17] Nagelkerke NJ, Jha P, de Vlas SJ, Korenromp EL, Moses S (2002). Modelling HIV/AIDS epidemics in Botswana and India: impact of interventions to prevent transmission.. Bull World Health Organ.

[18] Jha P, Nagelkerke NJD, Ngugi EN, Prasada Rao JVR, Willbond B (2001). Reducing HIV transmission in developing countries.. Science.

[19] Moses S, Blanchard JF, Kang H, Emmanuel F, Paul SR (2006). AIDS in South Asia: Understanding and Responding to a Heterogeneous Epidemic.. The International Bank for Reconstruction and Development/The World Bank.

[20] Steinbrook R (2007). HIV in India - a complex epidemic.. NEJM.

[21] Dandona L, Sisodia P, Kumar SG, Ramesh YK, Kumar AA (2005). HIV prevention programmes for female sex workers in Andhra Pradesh, India: outputs, cost and efficiency.. BMC Public Health Sep.

[22] Schneider JA, Saluja GS, Oruganti G, Dass S, Tolentino J (2007). HIV infection dynamics in rural Andhra Pradesh south India: a sexual-network analysis exploratory study.. AIDS Care.

[23] Chandrasekaran P, Dallabetta G, Loo V, Rao S, Gayle H (2006). Containing HIV/AIDS in India: the unfinished agenda.. Lancet Infect Dis.

[24] Blanchard JF, Halli S, Ramesh BM, Bhattacharjee P, Washington RG (2007). Variability in the sexual structure in a rural Indian setting: implications for HIV prevention strategies.. Sexually Transmitted Infections.

[25] Halli SS, Buzdugan R, Ramesh BM, Gurnani V, Sharma V (2009). Assessing HIV risk in workplaces for prioritizing HIV preventive interventions in Karnataka State, India.. Sexually Transmitted Diseases.

[26] Brahmam GN, Kodavalla V, Rajkumar H, Rachakulla HK, Kallam S (2008). Sexual practives, HIV and sexually transmitted infections among self-identified men who have sex with men in four high HIV prevalence states of India.. AIDS.

[27] Mahanta J, Medhi GK, Paranjape RS, Roy N, Kohli A (2008). Injecting and sexual risk behaviours, sexually transmitted infections and HIV prevalence in injecting drug users in three states in India.. AIDS.

[28] Panda S, Chatterjee A, Bhattacharya SK, Manna B, Singh PN (2000). Transmission of HIV from injection drug users to their wives in India.. International Journal of STD & AIDS.

[29] Sarkar K, Bal B, Mukherjee R, Chakraborty S, Niyogi SK (2006). Epidemic of HIV coupled with hepatitis C virus among injecting drug users of Himalayan West Bengal, Eastern India, Bordering Nepal, Bhutan, and Bangladesh.. Substance Use and Misuse.

[30] Hernandez AL, Lindan CP, Mathur M, Ekstrand M, Madhivanan P (2006). Sexual behavior among men who have sex with women, men and hijras in Mumbai, India - multiple sex risks.. AIDS and Behavior.

[31] National AIDS Control Organization (2007). HIV Sentinal Surveillance and HIV Estimation in India, 2007. A Technical Brief..

[32] Solomon SS, Srikrishnan AK, Sifakis F, Mehta SH, Vasudevan CK (2010). The Emerging HIV Epidemic among Men Who have Sex with Men in Tamil Nadu, India: Geographic Diffusion and Bisexual Concurrency.. AIDS Behav.

[33] Verma RK, Collumbien M (2004). Homosexual activity among rural Indian men: implications for HIV interventions.. AIDS.

[34] Hawkes S, Collumbien M, Platt L, Lalji N, Rizvi N (2009). HIV and other sexually transmitted infections among men, transgenders and women selling sex in two cities in Pakistan: a cross-sectional prevalence survey.. Sexually Transmitted Infections.

[35] Baral S, Sifakis F, Cleghorn F, Beyrer C (2007). Elevated risk for HIV infection among men who have sex with men in low- and middle-income countries 2000–2006: a systematic review.. PLoS Med.

[36] van Griensven F, de Lind van Wijngaarden JW, Baral S, Grulich A (2009). The global epidemic of HIV infection among men who have sex with men.. Current Opinion in HIV and AIDS.

[37] Parekh S (2003). Homosexuality in India: the light at the end of the tunnel.. Journal of Gay & Lesbian Mental Health.

[38] Phillips AE, Lowndes CM, Boily MC, Garnett GP, Gurav K (2010). Men who have sex with men and women in Bangalore, South India, and potential impact on the HIV epidemic.. Sex Transm Infect.

[39] Houston E, McKirnan DJ (2007). Intimate partner abuse among gay and bisexual men: risk correlates and health outcomes.. J Urban Health.

[40] Phillips AE, Boily MC, Lowndes CM, Garnett GP, Gurav K (2008). Sexual identity and its contribution to MSM risk behavior in Bangaluru (Bangalore), India: the results of a two-stage cluster sampling survey.. J LGBT Health Res.

[41] Ramesh BM, Moses S, Washington R, Isac S, Mohapatra B (2008). Determinants of HIV prevalence among female sex workers in four south Indian states: analysis of cross-sectional surveys in twenty-three districts.. AIDS.

[42] Saidel T, Adhikary R, Mainkar M, Dale J, Loo V (2008). Baseline integrated behavioural and biological assessment among most at-risk populations in six high-prevalence states of India: design and implementation challenges.. AIDS.

[43] Berrios DC, Avins AL, Haynes-Sanstad K, Eversley R, Woods WJ (1995). Screening for human immunodeficiency virus antibody in urine.. Arch Pathol Lab Med.

[44] Oelemann WM, Lowndes CM, Verissimo Da Costa GC, Morgado MG, Castello-Branco LR (2002). Diagnostic detection of human immunodeficiency virus type 1 antibodies in urine: a brazilian study.. Journal of Clinical Microbiology.

[45] Hilbe JM (2007). Negative Binomial Regression.

[46] StataCorp (2010). Stata Statistical Software: Release 11. 11.0 ed.

[47] UCLA: Academic Technology Services Statistical Consulting Group (2010).

[48] Beattie TSH, Bhattacharjee P, Ramesh BM, Gumani V, Anthony J (2010). Violence against female sex workers in Karnataka state, south India: impact on health, and reductions in violence following an intervention program.. BMC Public Health.

[49] Reddy G (2005). Geographies of contagion: Hijra, Kothis and the politics of sexual marginality in Hyderabad.. Anthropology & Medicine.

[50] Sahastrabuddhe S, Gupta A, Stuart E, Godbole S, Ghate M (2011). Sexually Transmitted Infections and Risk Behaviors Among Transgender persons (Hijras) of Pune, India.. J Acquir Immune Defic Syndr.

[51] Khan S, Aggleton P (1999). Through a window darkly: men who sell sex to men in India and Bangladesh.. Men who sell sex: International perspectives on male prostitution and HIV/AIDS.

[52] Jayasree AK (2004). Searching for Justice for Body and Self in a Coercive Environment: Sex Work in Kerala, India.. Reproductive Health Matters.

[53] Panchanadeswaran S, Johnson SC, Sivaram S, Srikrishnan AK, Latkin C (2008). Intimate partner violence is as important as client violence in increasing street-based female sex workers' vulnerability to HIV in India.. Int J Drug Policy.

[54] Reza-Paul S, Beattie T, Syed HU, Venukumar KT, Venugopal MS (2008). Declines in risk behaviour and sexually transmitted infection prevalence following a community-led HIV preventive intervention among female sex workers in Mysore, India.. AIDS.

[55] Shannon K, Kerr T, Allinott S, Chettiar J, Shoveller J (2008). Social and structural violence and power relations in mitigating HIV risk of drug-using women in survival sex work.. Soc Sci Med.

[56] Rhodes T, Simic M, Baros S, Platt L, Zikic B (2008). Police violence and sexual risk among female and transvestite sex workers in Serbia: qualitative study.. BMJ.

[57] Lorway R, Shaw SY, Hwang S, Reza-Paul S, Wylie J (2010). From individuals to complex systems: exploring the sexual networks of MSM in three cities in Karnataka, India.. Sexually Transmitted Infections In Press.

[58] Ramachandran S, Yonas MA, Silvestre AJ, Burke JG (2010). Intimate partner violence among HIV-positive persons in an urban clinic.. AIDS Care.

[59] Chandrasekaran P, Dallabetta G, Loo V, Mills S, Saidel T (2008). Evaluation design for large-scale HIV prevention programmes: the case of Avahan, the India AIDS initiative.. AIDS.

[60] Shaw SY, Emmanuel F, Adrien A, Holte-McKenzie M, Archibald CP (2011). The descriptive epidemiology of male sex workers in Pakistan: a biological and behavioural examination.. Sex Transm Infect.

