# Supplementary material for: Factors Associated with Sexual Violence against Men Who Have Sex with Men and Transgendered Individuals in Karnataka, India
Source: PLoS One. 2012 Mar 20;7(3):e31705. doi: 10.1371/journal.pone.0031705 (PMC3308942; doi:10.1371/journal.pone.0031705)
Supplement: Table S1 — Comparison of socio-demographic, sexual behaviour and health care access characteristics among men who have sex with men and transgenders (MSM-T) experiencing sexual violence in the last year, Karnataka, South India (2008). (DOC) [file pone.0031705.s001.doc]

**Table S1: Comparison of socio-demographic, sexual behaviour and health care access characteristics among men who have sex with men and transgenders (MSM-T) experiencing sexual violence in the last year, Karnataka, South India (2008)**^*^

|  | **Experiencing Violence % (n=95, 17.5%)** | **Not Experiencing Violence % (n=448, 82.5%)** |
| --- | --- | --- |
| **Socio-demographic characteristics** |  |  |
| Age |  |  |
| 18-24 | 42.6 | 27.9 |
| 25-29 | 27.6 | 18.4 |
| 30-39 | 21.4 | 27.7 |
| 40+ | 8.4 | 25.9 |
| *Missing* | *0.0* | *0.2* |
| *Mean (SE)* | *27.7 (0.9)* | *31.6 (0.6)* |
| Can read and write (%) |  |  |
| Yes | 66.4 | 71.8 |
| No | 33.6 | 28.0 |
| *Missing* | *0.0* | *0.2* |
| Marital status (%) |  |  |
| Currently married | 30.5 | 56.2 |
| Not currently married | 69.6 | 43.6 |
| *Missing* | *0.0* | *0.2* |
| Identity (%)^**^ |  |  |
| Bisexual/Panthi/Other | 5.5 | 15.1 |
| Double decker | 48.4 | 62.1 |
| Kothi/Hijra | 45.8 | 22.6 |
| *Missing* | *0.4* | *0.2* |
| District |  |  |
| Belgaum | 48.5 | 57.9 |
| Bellary | 19.5 | 11.7 |
| Shimoga | 20.5 | 26.0 |
| Mysore | 11.5 | 4.4 |
|  |  |  |
| **Sexual behaviour characteristics** |  |  |
|  |  |  |
| Never had vaginal intercourse | 53.8 | 32.5 |
| Had vaginal intercourse | 46.2 | 67.5 |
| Age at first vaginal intercourse (n=328) |  |  |
| <17 | 16.4 | 17.9 |
| 18-20 | 29.9 | 36.6 |
| 21-24 | 34.0 | 22.5 |
| 25+ | 15.3 | 22.4 |
| *Missing* | *4.4* | *0.6* |
| *Mean (SE)* | *20.9 (0.2)* | *20.9(0.2)* |
| Age at first anal intercourse |  |  |
| <17 | 58.8 | 41.6 |
| 18-20 | 23.1 | 36.5 |
| 21-24 | 8.5 | 11.1 |
| 25+ | 6.9 | 10.5 |
| *Missing* | *2.8* | *0.2* |
| *Mean (SE)* | *17.0 (0.6)* | *18.8 (0.3)* |
| Ever used condom in anal intercourse |  |  |
| Yes | 94.3 | 90.3 |
| No | 5.7 | 9.6 |
| *Missing* | *0.0* | *0.2* |
| Condom used in last anal intercourse |  |  |
| Yes | 91.4 | 81.5 |
| No | 2.0 | 8.6 |
| *Missing* | *6.6* | *10.9* |
| Wanted to use a condom but could not, l\past 6 months |  |  |
| Yes | 28.9 | 22.4 |
| No | 65.4 | 67.9 |
| *Missing* | *5.7* | *9.8* |
| Main reason for not using a condom (%, n=146) |  |  |
| Partner did not want to | 27.4 | 24.0 |
| Condom not available | 40.1 | 50.4 |
| Condom costs too much/condom broke/don’t like condoms | 12.6 | 17.3 |
| Other | 13.5 | 4.4 |
| *Missing* | *6.4* | *1.1* |
| Usual place of solicitation |  |  |
| Home | 9.7 | 15.8 |
| Public garden | 24.4 | 17.9 |
| Bus stop/stations | 31.7 | 29.0 |
| Other | 34.3 | 37.1 |
| *Missing* | *0.0* | *0.2* |
| Usual place for anal sex |  |  |
| Home | 11.6 | 16.2 |
| Public garden | 18.0 | 15.1 |
| Bus stop/stations | 0.4 | 0.8 |
| Other | 70.1 | 67.8 |
| *Missing* | *0.0* | *0.2* |
| Number of times had anal sex with regular male sex partners, past week (%) |  |  |
| 0 | 34.2 | 31.9 |
| 1-2 | 24.7 | 40.0 |
| 3-4 | 19.0 | 15.0 |
| 5+ | 22.1 | 12.9 |
| *Missing* | *0.0* | *0.2* |
| *Mean (SE)* | *3.3(0.9)* | *2.0 (0.2)* |
| Condom use with last regular male sex partner (%, n=381) |  |  |
| Yes | 91.4 | 88.9 |
| No | 8.6 | 11.1 |
| Number of times had anal sex with non-regular male sex partners, past week (%) |  |  |
| 0 | 34.3 | 44.3 |
| 1-2 | 33.8 | 38.4 |
| 3-4 | 19.4 | 10.6 |
| 5+ | 12.5 | 6.4 |
| *Missing* | *0.0* | *0.3* |
| *Mean (SE)* | *2.8(0.7)* | *1.3 (0.1)* |
| Condom use with last non-regular male sex partner (%, n=299) |  |  |
| Yes | 90.5 | 86.6 |
| No | 9.5 | 13.4 |
| Have a main male sexual partner |  |  |
| Yes | 57.3 | 37.1 |
| No | 42.7 | 62.7 |
| *Missing* | *0 (0.0)* | *1 (0.2)* |
| Ever received cash/gift for anal sex |  |  |
| Yes | 45.5 | 21.1 |
| No | 54.5 | 78.9 |
| Age at first paid sex (%, n=175) |  |  |
| < 15 | 7.6 | 2.5 |
| 15-19 | 60.7 | 58.7 |
| 20-24 | 28.4 | 25.7 |
| 25+ | 3.3 | 13.1 |
| *Mean (SE)* | 18.0 (0.4) | 19.5 (0.4) |
| **Health care access characteristics** |  |  |
| Number of general doctor visits, past one year (%, n=378) |  |  |
| 0-3 | 28.4 | 24.0 |
| 4-7 | 12.4 | 23.5 |
| 8-14 | 23.1 | 32.3 |
| 15+ | 36.2 | 20.2 |
| *Mean (SE)* | 12.5 (1.4) | 10.3 (0.6) |
| Number of STI-related doctor visits, past one year (%, n=376) |  |  |
| 0 | 56.7 | 66.7 |
| 1-2 | 23.8 | 16.2 |
| 3+ | 19.5 | 17.1 |
| *Mean (SE)* | 1.7 (0.4) | 1.5 (0.2) |
| Ever taken an HIV/AIDS test |  |  |
| Yes | 36.2 | 31.7 |
| No | 56.3 | 56.7 |
| *Missing* | *5 (7.6)* | *54 (11.7)* |
| Prevalence of Infections (%) |  |  |
| HIV +ve | 20.3 | 12.4 |
| HIV -ve | 77.6 | 86.0 |
| *Missing* | *2.1* | *1.7* |
| CT +ve | 0.0 | 0.9 |
| CT –ve | 86.4 | 92.0 |
| *Missing* | *13.6* | *7.0* |
| GC +ve | 0.0 | 0.4 |
| GC –ve | 86.4 | 92.6 |
| *Missing* | *13.6* | *7.0* |
|  |  |  |

^*^All percentages are weighted percentages, missing values reported where applicable

^**^Kothis: those who primarily practice receptive anal sex; Hijras: transgenders who often self-identify as female; Panthis: those who primarily practice insertive anal sex; Double-deckers: those who practice both insertive/receptive anal sex
