# Supplementary material for: Factors Associated with Sexual Violence against Men Who Have Sex with Men and Transgendered Individuals in Karnataka, India
Source: PLoS One. 2012 Mar 20;7(3):e31705. doi: 10.1371/journal.pone.0031705 (PMC3308942; doi:10.1371/journal.pone.0031705)
Supplement: Table S2 — Prevalence of violence by socio-demographic, sexual behaviour and health care access characteristics among men who have sex with men and transgenders (MSM-T) experiencing sexual violence in the last year, Karnataka, South India (2008). (DOC) [file pone.0031705.s002.doc]

**Table S2: Prevalence of violence by socio-demographic, sexual behaviour and health care access characteristics among men who have sex with men and transgenders (MSM-T) experiencing sexual violence in the last year, Karnataka, South India (2008)**^*^

|  | **Prevalence**  **of Violence (%)** | **P (weighted,**  **Wald test)^**^** |
| --- | --- | --- |
| **Socio-demographic characteristics** |  |  |
| Age |  | .002 |
| 18-24 | 20.9 |  |
| 25-29 | 20.6 |  |
| 30-39 | 11.8 |  |
| 40+ | 5.3 |  |
| Can read and write (%) |  | .344 |
| Yes | 13.8 |  |
| No | 17.2 |  |
| Marital status (%) |  | <.001 |
| Currently married | 8.6 |  |
| Not currently married | 21.6 |  |
| Identity (%)^***^ |  |  |
| Bisexual/Panthi/Other | 5.9 | <.001 |
| Double decker | 11.9 |  |
| Kothi/Hijra | 25.9 |  |
| District |  | .036 |
| Belgaum | 12.7 |  |
| Bellary | 22.4 |  |
| Shimoga | 12.0 |  |
| Mysore | 31.0 |  |
|  |  |  |
| **Sexual behaviour characteristics** |  |  |
|  |  |  |
| Never had vaginal intercourse | 22.3 | .002 |
| Had vaginal intercourse | 10.6 |  |
| Age at first vaginal intercourse (n=328) |  | .290 |
| <17 | 9.8 |  |
| 18-20 | 8.8 |  |
| 21-24 | 15.2 |  |
| 25+ | 7.5 |  |
| Age at first anal intercourse |  | .029 |
| <17 | 19.7 |  |
| 18-20 | 9.9 |  |
| 21-24 | 11.7 |  |
| 25+ | 10.1 |  |
| Ever used condom in anal intercourse |  | .367 |
| Yes | 15.3 |  |
| No | 9.3 |  |
| Condom used in last anal intercourse |  | <.001 |
| Yes | 16.3 |  |
| No | 2.2 |  |
| Wanted to use a condom but could not, past 6 months |  | .320 |
| Yes | 18.3 |  |
| No | 14.3 |  |
| Main reason for not using a condom  (%, n=146) |  | .612 |
| Partner did not want to | 20.3 |  |
| Condom not available | 15.1 |  |
| Condom costs too much/condom broke/don’t like condoms | 14.0 |  |
| Other | 29.8 |  |
| Usual place of solicitation |  | .406 |
| Home | 9.6 |  |
| Public garden | 19.0 |  |
| Bus stop/stations | 15.9 |  |
| Other | 13.8 |  |
| Usual place for anal sex |  | .567 |
| Home | 11.0 |  |
| Public garden | 17.1 |  |
| Bus stop/stations | 7.7 |  |
| Other | 15.2 |  |
| Number of times had anal sex with regular male sex partners, past week (%) |  | .088 |
| 0 | 15.7 |  |
| 1-2 | 9.7 |  |
| 3-4 | 18.0 |  |
| 5+ | 22.9 |  |
| Condom use with last regular male sex partner (%, n=381) |  | .669 |
| Yes | 14.8 |  |
| No | 11.6 |  |
| Number of times had anal sex with non-regular male sex partners, past week (%) |  | .043 |
| 0 | 11.8 |  |
| 1-2 | 13.2 |  |
| 3-4 | 24.0 |  |
| 5+ | 25.4 |  |
| Condom use with last non-regular male sex partner (%, n=299) |  | .507 |
| Yes | 17.7 |  |
| No | 12.7 |  |
| Have a main male sexual partner |  | .006 |
| Yes | 21.1 |  |
| No | 10.6 |  |
| Ever received cash/gift for anal sex |  | <.001 |
| Yes | 27.2 |  |
| No | 10.7 |  |
| Age at first paid sex (%, n=175) |  | .142 |
| < 15 | 52.9 |  |
| 15-19 | 27.5 |  |
| 20-24 | 28.9 |  |
| 25+ | 8.6 |  |
| **Health care access characteristics** |  |  |
| Number of general doctor visits, past one year (%, n=378) |  | .017 |
| 0-3 | 17.0 |  |
| 4-7 | 8.4 |  |
| 8-14 | 11.1 |  |
| 15+ | 23.8 |  |
| Number of STI-related doctor visits, past one year (%, n=376) |  | .393 |
| 0 | 12.7 |  |
| 1-2 | 20.1 |  |
| 3+ | 16.3 |  |
| Ever taken an HIV/AIDS test |  | . 690 |
| Yes | 16.5 |  |
| No | 14.7 |  |
| Infections |  |  |
| HIV +ve | 22.2 | .104 |
| HIV -ve | 13.5 |  |
| CT +ve | 0.0 | .407 |
| CT –ve | 15.0 |  |
| GC +ve | 0.0 | .549 |
| GC –ve | 14.9 |  |

^*^All percentages are weighted percentages; ^**^Missing values not included in statistical tests

^***^Kothis: those who primarily practice receptive anal sex; Hijras: transgenders who often self-identify as female; Panthis: those who primarily practice insertive anal sex; Double-deckers: those who practice both insertive/receptive anal sex
