# Supplementary material for: Factors Associated with Sexual Violence against Men Who Have Sex with Men and Transgendered Individuals in Karnataka, India
Source: PLoS One. 2012 Mar 20;7(3):e31705. doi: 10.1371/journal.pone.0031705 (PMC3308942; doi:10.1371/journal.pone.0031705)
Supplement: Table S3 — Crude (OR) and Adjusted odds ratios (AOR) and 95% confidence intervals (95%CI) from weighted logistic regression models examining correlates of sexual violence among men who have sex with men and transgenders (MSM-T), by total sample and stratified by sex work status, Karnataka, South India (2008). (DOC) [file pone.0031705.s003.doc]

**Table S3: Crude (OR) and Adjusted odds ratios (AOR) and 95% confidence intervals (95%CI) from weighted logistic regression models examining correlates of sexual violence among men who have sex with men and transgenders (MSM-T), by total sample and stratified by sex work status, Karnataka, South India (2008)^*^**

|  | **Prevalence of**  **Violence (%)** | **OR** | **95% CI** | **AOR** | **95% CI** | **P value^**^** |
| --- | --- | --- | --- | --- | --- | --- |
| **Total Sample (N=543)** |  |  |  |  |  |  |
| Age (mean) | 27.2 | 0.94 | (0.91,0.97) | 0.94 | (0.91,0.97) | .001 |
| Identity^***^ |  |  |  |  |  |  |
| Bisexual/Panthi/Other | 5.9 | *Ref* | *--* | *Ref* | *--* | *--* |
| Double decker | 11.9 | 2.16 | (0.78,5.95) | 2.22 | (0.84,5.87) | .106 |
| Kothi/Hijra | 25.9 | 5.60 | (1.90,16.46) | 3.51 | (1.16,10.59) | .027 |
| Currently Married |  |  |  |  |  |  |
| No | 21.6 | *Ref* | *--* | *Ref* | *--* | *--* |
| Yes | 8.6 | 0.34 | (0.20,0.59) | 0.83 | (0.45,1.53) | .547 |
| Have a main male sex partner |  |  |  |  |  |  |
| No | 10.6 | *Ref* | *--* | *Ref* | *--* | *--* |
| Yes | 21.1 | 2.27 | (1.27,4.06) | 1.65 | (0.91,3.00) | .098 |
| District |  |  |  |  |  |  |
| Belgaum | 12.7 | *Ref* | *--* | *Ref* | *--* | *--* |
| Bellary | 22.4 | 1.98 | (0.95,4.16) | 1.28 | (0.49,3.33) | .602 |
| Shimoga | 12.0 | 0.94 | (0.39,2.29) | 0.85 | (0.31,2.36) | .756 |
| Mysore | 31.0 | 3.09 | (1.61,5.92) | 1.80 | (0.77,4.20) | .171 |
| Number of non-regular male sex partners, past week |  |  |  |  |  |  |
| 0 | 11.8 | *Ref* | *--* | *Ref* | *--* | *--* |
| 1-4 | 15.8 | 1.40 | (0.81,2.45) | 1.35 | (0.70,2.60) | .363 |
| 5+ | 25.4 | 2.55 | (1.28,5.05) | 1.80 | (0.96,3.50) | .067 |
| HIV status |  |  |  |  |  |  |
| Negative | 13.5 | *Ref* | *--* | *Ref* | *--* | *--* |
| Positive | 22.2 | 1.82 | (0.87,3.81) | 2.02 | (0.82,4.97) | .123 |
|  |  |  |  |  |  |  |

^*^All counts are unweighted counts; all percentages are weighted percentages; ^**^P-value reported for adjusted analyses only

^***^Kothis: those who primarily practice receptive anal sex; Hijras: transgenders who often self-identify as female; Panthis: those who primarily practice insertive anal sex; Double-deckers: those who practice both insertive/receptive anal sex
